# Supplementary material for: What are the impacts of increasing cost-effectiveness Threshold? a protocol on an empirical study based on economic evaluations conducted in Thailand
Source: PLoS One. 2022 Oct 3;17(10):e0274944. doi: 10.1371/journal.pone.0274944 (PMC9529087; doi:10.1371/journal.pone.0274944)
Supplement: Attachment — Submitted filename: Comment_Study Protocol CET.docx [file pone.0274944.s001.docx]

**Comment:**

This study protocol entitles “What are the impacts of increasing cost-effectiveness threshold? A protocol on and empirical study based on economic evaluations conducted in Thailand.” Overall, the protocol is interesting and the future findings of the study would be of interest and benefit to Thailand and other countries. Several comments are as follows:

1. Authors should provide reasons why the CET of Thailand has been revised twice within 5-6 years (2008-2013) and constant up until present (2014-2022)? What were the driven factors to specify the amount of CET of Thailand?
2. Based on the conceptual framework (Figure 1), three variables, drug price, probability of high-cost drug, budget impact, will be measured following to the objectives of the study. How do the authors plan to measure the access to medications & overall population health? Should it be one of main objective similar to the other 3 objectives?
3. For the extreme scenario with undesirable outcomes from the conceptual framework, the budget impact might not necessarily increase when an access to medication decreases? BIA is the product of cost and population. Although drug price increases, the population uptake reduces, it is possible to have same/lower/higher budget.
4. Table 1 shows summary of confounders. Do authors consider GDP/GNI or country income as the confounder? It should impact on CET based on WHO guideline of cost-effectiveness.
5. Equation 1 and equation 2, the term “Z” refers to all 6 groups of potential confounders?
6. The process of NLEM consideration might take time which would be different in each country. The CET was revised twice during the year of 2008-2013. The drug was submitted at one year, like 2008, but study was done and considered into NLEM at another year, like 2010. Which year will be used for analysis?
