# Supplementary material for: What are the impacts of increasing cost-effectiveness Threshold? a protocol on an empirical study based on economic evaluations conducted in Thailand
Source: PLoS One. 2022 Oct 3;17(10):e0274944. doi: 10.1371/journal.pone.0274944 (PMC9529087; doi:10.1371/journal.pone.0274944)
Supplement: Attachment — Submitted filename: ResponseToReviewers_PONE-D-22-01139R1_30Aug22.docx [file pone.0274944.s003.docx]

**Response to Reviewer: Manuscript ID: PONE-D-22-01139R1**

What are the Impacts of Increasing Cost-effectiveness Threshold? A Protocol on an Empirical Study based on Economic Evaluations conducted in Thailand

**Independent Review Report, Reviewer 1**
**EVALUATION**

Reviewer #1: The authors have addressed the comments made satisfactorily and have made changes to the manuscript accordingly. No further comments.

**RESPONSE:** Thank you.

**Independent Review Report, Reviewer 2
EVALUATION**
Reviewer #2: The revised version is much clearer. However, there are few points to clarify and consider.

1. In the introduction part, please consider add the recent study, which found that the empirical CET might be less than 1 GDP per capita (see, Gloria MAJ, Thavorncharoensap M, ChaikledkaewU, Youngkong S, Thakkinstian A, Culyer AJ. A Systematic Review of Demand-Side Methods of Estimating the Societal Monetary Value of Health Gain. Value Health. 2021 Oct;24(10):1423-1434)

**RESPONSE:** We added the highlighted reference accordingly. Thank you.

2. Impact on population health were not included in the analysis so please note in the conceptual framework figure.

**RESPONSE:** We added in the Figure that impact on population health was not included in the analysis.

3. Table 1:

• Variables that in the reference columns filled with “for information” are not confounders. Please take them out from table 1 as table 1 provides the list of confounders.

**RESPONSE:** Noted and we revised Table 1 accordingly.

• It seemed that if the type of variable was mentioned as “text” (e.g. primary endpoint, ICD), it will not be in the regression model. If so, they are not confounders as well. If they are not confounders please take them out from table 1.

**RESPONSE:** Noted and we revised Table 1 accordingly.

• Age of the patient – if it is the group, it should not be continuous variable

**RESPONSE:** We revised the variable type with explanation that age of the patient will be grouped to be a categorical variable; the exact categories will be determined after the review of included studies. We will know more once we review the studies.

• Burden of disease- should it be categorical variable instead of continuous variable (e.g., < xxx,xxx-xxx, etc.)

**RESPONSE:** We revised accordingly. Thank you.

• For variable “publication”, any publication will be counted or only effectiveness, cost-effectiveness?

**RESPONSE:** Only the cost-effectiveness studies will be counted.

4. Table 2: Details of 10 health economic assessment … should added “ that were conducted by pharmaceutical company”

**RESPONSE:** We revised the table accordingly. Thank you.

5. Page 11: Under equation 2. For description of ICER it , Z ij, whether the word “included” should be taken out. Please change Zij to Zit

**RESPONSE:** We removed the word “included” for both ICER it and Z it.
